# Harmonization of medical products regulation: a key factor for improving regulatory capacity in the East African Community

**DOI:** 10.1186/s12889-021-10169-1

**Published:** 2021-01-21

**Authors:** Margareth Ndomondo-Sigonda, Jacqueline Miot, Shan Naidoo, Nelson E. Masota, Brian Ng’andu, Nancy Ngum, Eliangiringa Kaale

**Affiliations:** 1 Pharmacology Division, Department of Pharmacy and Pharmacology, Faculty of Health Sciences, University of Witwatersrand, Johannesburg, South Africa; 2 African Union Development Agency -New Partnership for Africa’s Development (AUDA-NEPAD), Midrand, South Africa; 3 Health Economics and Epidemiology Research Office, Department of Internal Medicine, School of Clinical Medicine, Faculty of Health Sciences, University of Witwatersrand, Johannesburg, South Africa; 4 Public Health Medicine Department, School of Public Health, University of Witwatersrand, Johannesburg, South Africa; 5 Institute for Pharmacy and Food Chemistry, University of Wuerzburg, Wuerzburg, Germany; 6 School of Pharmacy, Muhimbili University of Health and Allied Sciences, Dar es Salaam, Tanzania

**Keywords:** East African community, Medical products regulation, Regulatory capacity, Africa regulatory harmonization, Registration, Regulatory inspection, Efficiency, Governance

## Abstract

**Background:**

Limited capacity to regulate medical products is associated with circulation of products which do not meet standards of quality, safety and efficacy with negative public health and economic outcomes. This study focused on assessing the effect of the East African Community (EAC) medicines regulatory harmonization initiative on the capacity of national medicines regulatory agencies, with a focus on registration and inspection systems.

**Methods:**

An exploratory mixed-method design using both qualitative and quantitative data to access data from six national medicines regulatory authorities (NMRAs) and the EAC Secretariat. Data was collected using a combination of semi-structured interviews, questionnaires, and checklists for the period 2010/11–2015/16 with 2010/11 data serving as baseline. Heads of NMRAs, regulatory and monitoring and evaluation experts, and the EAC Secretariat Project Officer were enrolled in the study. A set of 14 indicators grouped into 6 categories were used to assess NMRAs performance.

**Results:**

Policy and legal frameworks provide a foundation for effective regulation. Collaboration, harmonization, joint dossier reviews and inspections of manufacturing sites, reliance and cooperation are key factors for building trust and capacity among NMRAs. Five out of six of the EAC Partner States have comprehensive medicines laws with autonomous NMRAs. All the NMRAs have functional registration and good manufacturing practice inspection systems supported by regional harmonised guidelines for registration, inspection, quality management and information management systems with four NMRAs attaining ISO 9001:2015 certification.

**Conclusions:**

The EAC regulatory harmonization initiative has contributed to improved capacity to regulate medical products. The indicators generated from this research can be replicated for evaluation of similar initiatives across and beyond the African continent and contribute to public health policy.

## Background

The African continent constitutes 15% of the world’s population with a disproportionate disease burden of more than 25% [1, 2]. Africa’s high disease burden and high mortality from preventable and curable diseases are partly due to inadequate health systems, scarce financial and human resources as well as unavailability of and unaffordable medicines that are of good quality, safe and efficacious [3]. Lack of access to quality, safe, efficacious and affordable medicines is in part attributed to limited local pharmaceutical manufacturing base and weak medicines regulatory systems [3]. In 2005 the Heads of State and Government of the African Union (AU) mandated formulation of the Pharmaceutical Manufacturing Plan for Africa (PMPA) with the view of strengthening Africa’s ability to produce high quality and affordable medicines that will contribute to improved health and economic outcomes [4].

The coming into force of the African Continental Free Trade Area (AfCFTA) in 2019 serves as an impetus to boost intra-African trade through a single market of 1.2 billion people and a cumulative Gross Domestic Product (GDP) of over $3.4 trillion across the 55 member states of the AU [5, 6]. An estimated $259 billion health care and wellness sector by 2030 and the $45 billion projection African pharmaceutical market by 2020 are attributed to the changing economic profiles, rapid urbanization, increased healthcare spending and investment, and increasing incidence of chronic lifestyle diseases [7, 8]. The increasing demand of medicines in Africa due to population and economic growth as well as raising consumer awareness warrants governments’ investment in local production and effective regulation of medical products [6, 9].

For many years the capacity to regulate medicines in Sub-Saharan African countries has been confronted with fragmented legal frameworks, weak management structures and processes, and a severe lack of staff and resources. This led to subsequent proliferation of substandard and falsified medicines in various markets [10, 11]. A report in 2010 revealed that 7% of the 46 sub-Saharan African countries have moderately developed medicine regulatory capacity, about 63% have minimal capacities and the remaining 30% have an NMRA in place [12]. Poor inspection practices, ineffective licensing and product registration systems, inadequate access to quality control laboratories, non-existent pharmacovigilance, clinical trials oversight and drug promotion control systems, with subsequent 30% product quality failure rates are characteristic of regulatory systems in Africa [13]. This is coupled with inadequate communication and information exchange systems, lack of transparency and accountability and conflict of interest. Another study conducted in 2017 revealed that except for Sahrawi Republic, the remaining African countries have NMRAs with varying organizational setups and levels of functionality, operating either as units or departments within Ministries of Health, or as semi- or fully autonomous agencies [14].

Governments and partners are called to build and strengthen medicines regulatory systems with appropriate legal frameworks and institutions so as to carry out regulatory functions while supported by mechanisms for collaboration among agencies [11]. Effective and efficient regulation of medical products provides an opportunity for investment in manufacturing, trade, and sale of pharmaceutical products, as well as an increase in research and development of new medical products and technologies. In turn, these yield social and economic benefits to the patients and communities at large [4, 13, 14]. Building medical products regulatory capacity is also crucial for achieving Universal Health Coverage (UHC) goals, the AU Agenda 2063 aspirations and goals 1 and 3, as well as Sustainable Development Goals (SDGs) on access to quality, safe and efficacious health products to the people [15].

The African Medicines Regulatory Harmonization (AMRH) Initiative is an attempt by the AU to strengthen regulatory capacity, encourage harmonization of regulatory requirements and expediting access to good quality, safe, and effective medicines [16]. The initiative is implemented as part of the AU’s PMPA, a policy framework to provide an enabling regulatory environment for local production and contribute to the UHC, AU Agenda 2063 and SDGs goals [17, 18].

For the period between 2015 and 2016, countries in the East African Community (EAC) have recorded significant improvements in registration timelines from an average of 2 to 7 years to a median of 7 months [19, 20]. Within the *Zazibona*, the median timeline to product recommendation was 5 months in 2014 and 9 months for three consecutive years between 2015 and 2017. *Zazibona* is a collaborative procedure for registration of medicines initiated by Zambia, Zimbabwe, Botswana and Namibia in the Southern African Development Community SADC region.

The AMRH initiative is as well implemented in the Economic Community of West African States (ECOWAS), Intergovernmental Authority on Development (IGAD) and Economic Community of Central African States (ECCAS). It covers more than 85% of the Sub-Saharan African countries which are at different levels of its implementation. In order to address the problem of non-coherent medicines laws in African countries, the AMRH Initiative developed a Model Law on medical products regulation so as to ensure effective regulation and promotion of harmonization [20, 21]. The Model Law which among others promotes the establishment of autonomous agencies was adopted by the AU Assembly in January 2016 and has been domesticated by more than 12 AU Member States. Since 2014, eleven regional centers of regulatory excellence have been designated since 2014 to provide coordinated and structured regulatory science training programmes using the existing academic institutions in partnership with regulatory agencies [20].

Understanding the relevance of various regulatory interventions undertaken at country, regional and continental levels is important for informing regulatory policy reforms undertaken by the AU, governments, and partners [22]. For this reason, this study was done to evaluate the effect of medicines regulatory harmonization initiative in the EAC region and to provide insights on whether it is making an impact on national regulatory capacity. We aimed at determining factors related to the improving regulatory capacity in the EAC NMRAs. Specifically; to determine the level at which countries have: i) Implemented agreed Common Technical Document (CTD) for registration of medicines in the EAC Partner States. ii) Implemented common Information Management Systems (IMS) for medicines registration in each of the EAC Partner States’ NMRAs, and whether or not the IMS is linked in all Partner States and EAC Secretariat. iii) Implemented Quality Management Systems (QMS) in each of the EAC Partner States’ NMRAs. iv) The level of capacity attained at regional and national levels in implementing medicines registration harmonization in the EAC region. v) Whether or not a framework for mutual recognition procedure has been developed and implemented among the EAC Partner States, and lastly vi) Whether or not a platform for information sharing with key stakeholders at national and regional level has been established. The hypothesis is that the EAC MRH Project implemented under the AMRH Initiative has increased regulatory capacity in the EAC Partner States.

## Methods

Fourteen (14) indicators were developed and grouped into 6 main categories to ensure objective assessment of performance of regional medicines regulatory harmonization initiatives, as indicated in the indicators matrix (Table 1) [23]. Each indicator was further refined and validated to facilitate users’ understanding, through validation workshops with monitoring and evaluation focal points from the EAC NMRAs, Regional Economic Communities (RECs) and the World Health Organization (WHO). The exercise aimed to ensure accuracy of data collected and to enable objective analysis.

**Table 1:** Indicators Matrix

	Indicator Title	Type	Reporting Level	Frequency	Data Sources
**Category 1**	**Policy and legal framework**				
Indicator 1	National Medicines Policy (NMP)	Input	NMRA	Three yearly	WHO NMRA GBT, Government gazette, Ministry of Health
Indicator 2	Legal framework governing the regulation of medical products	Input	NMRA	Three yearly	WHO NMRA GBT, Government Gazette, National Law, Ministry of Justice library, NMRA records
**Category 2**	**NMRA governance**				
Indicator 3	NMRA level of autonomy	Output	NMRA	Three yearly	WHO NMRA GBT, CIRS OpERA programme, Government gazette, NMRA Financial report, Governance structure
Indicator 4	Availability of structures to support NMRA decision making process	Process	NMRA	Three yearly	NMRA Organisation/governance structure, Medicines law
**Category 4**	**Medicines evaluation and registration, and good manufacturing practice (GMP) inspection systems**				
Indicator 5	Availability of guidance and procedures for registration of medicines	Process	NMRA	Annually	WHO NMRA GBT, CIRS OpERA programme, NMRA records, Government policies and legislation
Indicator 6	NMRAs using regionally harmonized guidelines for product registration	Output	Regional	Annually	CIRS OpERA programme, NMRA Records; REC Records
Indicator 7	Availability of a process to track product registration applications and timelines	Process	NMRA	Annually	WHO NMRA GBT, CIRS OpERA programme, NMRA records
Indicator 8	Number of products applications with registration decisions per annum	Outcome	Regional	Annually	CIRS OpERA programme, NMRA records
Indicator 9	Proportion of NMRAs participating in joint assessments	Output	Regional	Annually	CIRS OpERA programme, REC records
Indicator 10	Proportion of product applications jointly assessed/ reviewed at regional level	Outcome	Regional; continental	Annually	CIRS OpERA programme, REC records
Indicator 11	Availability of a Good Manufacturing Practice (GMP) inspection guidance and procedure	Process	NMRA	Annually	WHO NMRA GBT, NMRA records, Government policies and legislation, WHO Assessment Reports
**Category 5**	**Functional Quality Management Systems (QMS)**				
Indicator 12	Implementation of Quality Management System (QMS) requirements by NMRA	Process	NMRA; regional	Annually	CIRS OpERA programme, NMRA QMS Records
Indicator 13	Percentage of NMRAs ISO 9001: 2015 Certification	Output	NMRA; regional	Annually	NMRAs QMS Records
**Category 6**	**Information Management Systems (IMS)**				
Indicator 14	Implementation of requirements for an integrated IMS	Output	NMRA; regional	Annually	WHO NMRA GBT, NMRA IMS

### Study design

In order to access data from six National Medicines Regulatory Authorities (NMRAs) and the EAC Secretariat, we employed an exploratory mixed-method design. We used Semi-structured interviews, questionnaires and checklists to gather data between 2010/11 and 2015/16, with 2010/11 data acting as the benchmark [24, 25]. During this period, both annual and three yearly data were gathered. A pilot situational analysis study informed the designing of the data collection tools [24, 26, 27]. In addition, we adopted other questions from the WHO Global Benchmarking Tool (WHO-GBT) for the Assessment of National Regulatory Frameworks [28]. We validated the data collection tools by pre-administering them some of the prospective respondents in all studied NMRAs.

During the first round of data collection, the Head and one monitoring and evaluation expert in each NMRA, as well as the project officer of the EAC MRH project responded to self-administered questionnaires and checklists [25]. We selected the respondents based on their roles and involvement in medicines policies and regulatory activities. The second round involved the conduction of semi-structured face-to-face interviews to one staff from medicines registration, GMP inspections, legal affairs, human resource and finance departments in addition to the previous set of respondents. One to two respondents were involved in each 1 to 2 h long interview session [24].

An acceptance letter and an interview subject guide were sent to the interviewees in advance. Moreover, we obtained a permission to record the responses in form of selected written notes from each respondent. Follow-up visits were carried out to capture the incomplete data and verify the previously obtained data. Confidentiality of all respondents was highly observed thought the study [24].

### Data analysis

Qualitative data analysis and interpretation was carried out on all indicators involving the availability and corresponding details of the National policies, laws, guidelines, provisions, legal frameworks, level of autonomy, references and regulatory standards, GMP guidance and procedures as well as status of implementation of different modules. Qualitative data obtained using questionnaires, interviews and desk reviews of documents was organized, analysed and evaluated manually with the help of tables on MS Excel®.

On the other hand, quantitative data on aspects related to numbers of applications, joint assessments, products registered, timelines to registration of products, and number of NMRAs under a particular aspect were tabulated and analysed using MS Excel® for means, standard deviations, frequencies and proportions. Where only a single point/year data was provided by the respondent(s), it was reported as such. Due to lack of data from some agencies in some of the studied aspects, only the available data was used in statistical analysis.

## Results

This publication is a continuation from a previous paper which looked at factors affecting financial sustainability of NMRAs in the EAC region [24]. For the purpose of this paper, findings are categorised into five main categories namely policy and legal framework, NMRA governance, medicines registration and good manufacturing practice (GMP) systems, QMS and IMS.

### Policy and legal frameworks

Progress has been observed in National Medicines Policies (NMPs) and Medicines Laws by the EAC Partner States as exemplified in Table 2. While only three countries indicated the availability of NMPs during the baseline study, currently all six countries have NMPs with a clear vision and indications for establishment of a semi- or autonomous NMRA. With respect to medicines laws, Tanzania-Mainland and Zanzibar amended their national laws in 2014 and 2016 respectively, while the Rwanda Food and Drugs Law was enacted in 2013 [29–34].

**Table 2:** Status of indicators under policy and legal frameworks category (2010/11–2015/16)

	National Medicines Regulatory Agency					
	Burundi	Kenya	Rwanda	Tanzania	Uganda	Zanzibar
**Indicator 1: National Medicines Policy (NMP)**						
Availability of a National Medicines Policy (NMP)	Yes	Yes	Yes^Δ^	Yes	Yes	Yes^Δ^
Year of approval of the NMP by the Cabinet of Ministers	2012	2012	2016^sd^	2007	2015	2014
Availability of a provision in the NMP for establishment of an autonomous national medicines regulatory agency (NMRA)	Yes	Yes	Yes^Δ^	Yes	Yes	Yes^Δ^
Existence of a vision for the NMRA	Yes	Yes	Yes^Δ^	Yes	Yes	Yes^Δ^
Policy comprehensiveness	Yes	Yes	Yes^Δ^	Yes	Yes	Yes^Δ^
**Indicator 2: Legal framework governing the regulation of medical products**						
Legal framework availability	Yes	Yes	Yes^Δ^	Yes	Yes	Yes^Δ^
Availability of a Law for regulating medicines in your country	Yes	Yes	Yes^Δ^	Yes	Yes	Yes^Δ^
Year of enactment of medicines law	1980	1957 (as amended in 2009)	2013	1978, (repealed 2003 and amended in 2004 & 2014)	1993	1986 (repealed in 2006 & amended in 2016)
Legislation comprehensiveness	No	–	Yes^Δ^	Yes	Yes	Yes^Δ^

NB: - = No data available/submitted; ^sd^ = Secondary data from the EAC MRH Project SC Meeting Report (2018); Yes^Δ^ = a change from No at baseline

### Governance

A baseline study revealed that, the Tanzania Medicine and Medical Devices Authority (TMDA), the Zanzibar Food and Drugs Board (ZFDB) and the Kenya Pharmacy and Poisons Board (PPB) were semi-autonomous entities, and that the head of the PPB also served as a Chief Pharmacist combining both regulatory and policy roles as the technical arm of the Ministry of Health. For Rwanda and Burundi, the respective Ministries of Health Departments administered regulatory functions. Moreover, for the TMDA, PPB and the Uganda National Drug Authority (NDA), the agencies had powers to charge fees for regulatory services and received very little or no government subvention [26, 27].

Results have shown a significant improvement in the level of autonomy of the NMRAs. Currently, four NMRAs are operating as semi-autonomous agencies namely: the Rwanda Food and Drugs Authority (RFDA), TMDA, Zanzibar Food and Drugs Authority (ZFDA), and PPB; while NDA operates as a fully autonomous agency. Furthermore, the National Pharmaceuticals Regulation Law of Burundi was under consideration by the Burundian Parliament, provides for the establishment of a semi-autonomous NMRA to be called the Drug and Food Regulatory Authority of Burundi (ABREMA) [35]. Table 3 provides analysis of the NMRAs governance framework including the level of autonomy.

**Table 3:** Status of indicators NMRA governance category (2010/11–2015/16)

	National Medicines Regulatory Agency					
	Burundi	Kenya	Rwanda	Tanzania	Uganda	Zanzibar
**Indicator 3: NMRA level of autonomy**						
NMRA’s level of autonomy	Department within the Ministry of Health	Semi-Autonomous	Semi-Autonomous	Semi-Autonomous	Autonomous	Semi-Autonomous
**Indicator 4: Availability of structures to support NMRA decision making process**						
Medicine Law	Republic of Burundi. Decret No. 100/150 du 30 September 1980 portant Organization de I’exercise de la Pharmacie au Burundi. 1980.	Republic of Kenya. The Pharmacy and Poisons Act, Chapter 244. 1957, as amended in 2009.	Republic of Rwanda. Law No. 47/2012 of 14/01/2013 relating to the Regulation and Inspection of Food and Pharmaceutical Products. 2013	United Republic of Tanzania (Mainland). Tanzania Food, Drugs and Cosmetics Act, Cap 219. 2003, as amended in 2004, 2014 & 2019	Republic of Uganda. The National Drug Policy and Authority Act. 1993.	United Republic of Tanzania (Zanzibar). The Zanzibar Food, Drugs and Cosmetics Act. No. 2 of 2006 as amended in 2016
Existence of NMRA Governing Board	No	Yes	Yes	Yes	Yes	Yes^Δ^

NB: Yes^Δ^ = a change from No at baseline

**Fig. 1:**
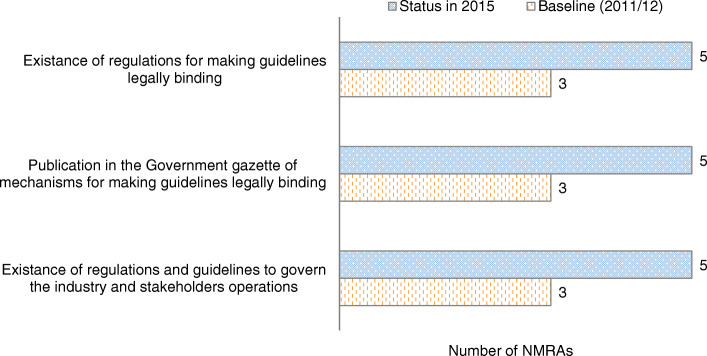
Trends in developing regulations and guidelines (Indicator 4) in the EAC Partner States (excluding Burundi) (2011/12–2014/15)

Moreover, there was a progressive trend in the development of regulations and guidelines among the EAC Partner States **(**Fig. 1). While baseline data showed three out of the six NMRAs had regulations and guidelines, the end-line data, shows five out six NMRAs reported having regulations and guidelines in place.

### Medicines registration system

Comparison of baseline data show improvement in registration systems in Rwanda and Burundi. All six NMRAs currently have a legal mandate to register medicines and a system to manage applications from receipt of a dossier to the issuance of a marketing authorization. In addition, all the NMRAs use the EAC harmonized guidelines for registration and Standard Operating Procedures (SoPs) for joint review of dossiers (Table 4). Also, all NMRAs participate in the EAC joint review of dossiers. However, the time taken to register a product based on the outcome of the joint review process still varies from country to country. For instance, out of fifteen applications received through the regional procedure, the TMDA registered all the products, whereas the proportion of products registered by other NMRAs was as shown in the brackets: PPB (13/15), Burundi (1/15), RFDA (9/15), ZFDA (1/15), and NDA (7/15) (Table 4). A reliance mechanism (where an agency relies on others in making a regulatory decision) exists in Kenya, Tanzania-Mainland, Tanzania-Zanzibar and Uganda while it was not the case for Burundi and Rwanda. Results on number of products applications received per annum (Table 4**,** under indicator 8) indicated the highest five years average of 800 new applications were received by the TMDA, followed by NDA (458), Burundi (70) and ZFDA (16). The PPB and RFDA indicated to have received 1030 and 833 applications respectively in 2016.

**Table 4:** Medicines Registration and GMP Inspection Systems in the EAC Partner States NMRAs (2011/12–2015/16)

	National Medicine Regulatory Agency					
	Burundi	Kenya	Rwanda	Tanzania	Uganda	Zanzibar
**Indicator 5: Availability of guidance and procedures for registration of medicines**						
Legal mandate to register medicines	Yes	Yes	Yes	Yes	Yes	Yes
Availability of a system for receiving applications, evaluating and providing marketing authorisation for medicines	Yes	Yes	Yes	Yes	Yes	Yes
**Indicator 6: NMRAs using regionally harmonized guidelines for product registration**						
Availability and use of EAC Harmonized guidelines for registration of medicines	Yes^Δ^	Yes^Δ^	Yes^Δ^	Yes^Δ^	Yes^Δ^	Yes^Δ^
Year EAC Harmonized Guidelines for Registration of Medicines came into force	2015	2015	2015	2015	2015	2015
**Indicator 7: Availability of a process to track product registration applications and timelines**						
Availability of mechanism for tracking registration timelines	No	Yes (2011)	No	Yes (2011)	No	Yes (2015)
Average timelines range attained for Fast-tracked products	–	3 months	–	4–6 months	–	–
Average timeline range attained for normal review	–	12 months*	–	12 months*	–	12 months*
**Indicator 8: Number of products applications with registration decisions per annum (Mean ± SD)**						
Applications received per annum	70.0 ± 42.0	1030*	833*	799.7 ± 275.2	457.80 ± 148.73	16.00 ± 18.89
Application carried over from previous reference year(s)	0.0 ± 0.0	1000*	575*	443.0 ± 301.4	–	0
Medicines registered by the NMRA per annum	0.0 ± 0.0	514*	175*	463.0 ± 224.6	344.40 ± 243.87	6.20 ± 4.76
**Indicator 9: Proportion of NMRAs participating in joint assessments**						
Participation in EAC joint assessments	Yes (2015)	Yes (2015)	Yes (2015)	Yes (2016)	Yes (2015)	Yes (2015)
Number of joint assessments participated by NMRA	1	1	1	3	1	3
Existence of policy on abridged procedure for registration of medicines	No	No	No	Yes (2011)	Yes (2016)	No. It is happening but there is no written policy yet.
Reference regulatory standard used on abridged procedure for registration of medicines	None	WHO-PQ	EAC & WHO-PQ	In-house SOP	EAC, SRAs & WHO-PQ	WHO-PQ
**Indicator 10: Proportion of product applications jointly assessed/ reviewed at regional level**						
Number of products registered based on EAC joint dossier review^sd^	1/15	13/15	9/15	15/15	7/15	1/15
Time taken to register medicines based on joint review outcome^sd^	–	–	–	–	–	–
**Indicator 11: Availability of a Good Manufacturing Practice (GMP) inspection guidance and procedure**						
Legal mandated to conduct GMP inspection	No	Yes (2011)	Yes (2015)	Yes (2011)	Yes (2011)	Yes (2015)
NMRA using EAC Harmonized guidelines for good manufacturing practice (GMP) inspections	No	Yes (2015)	Yes (2015)	Yes (2015)	Yes (2015)	Yes (2015)
Year EAC GMP inspection guidelines came into force	–	2015	2015	2015	2015	2015
Participating in EAC joint GMP inspections	Yes (2014&16)	Yes (2015)	Yes (2015)	Yes (2015)	Yes (2012)	Yes (2012)
Availability of policy on GMP assessment of pharmaceutical manufacturing sites using document review	No	No	Yes	No	Yes	No (it is happening but there is no written policy yet)
Reference regulatory standard used for GMP document review	–	–	EAC, PICs & WHO-PQ	–	EAC, WHO-PQ, US-FDA, EMA, PICS Inspection Reports	EAC GMP inspection report, WHO-Prequalification GMP inspection reports-

NB: Year in the bracket indicating the time from which the indicator started to apply; Yes^Δ^ = a change from No at baseline; * = only 2015/16 data (not average); − = No data available/ submitted; ^sd^ = Secondary data from the EAC MRH Project SC Meeting Report (2018)

### GMP inspection system

All six NMRAs have a legal mandate to conduct GMP inspection (Table 4, indicator 11). Except for Burundi NMRA, other NMRAs reported to be conducting inspections of manufacturing sites. While Kenya, Tanzania and Uganda NMRAs had conducted inspections since during the baseline study, Rwanda and Zanzibar NMRAs started in 2015. Moreover, All NMRAs indicated to be using the EAC harmonized guidelines for GMP inspection from 2015 when they came into force. Each NMRA reported having participated at least once in the EAC joint inspections. Additionally, the RFDA, NDA and ZFDA employ a reliance models by using GMP inspection reports from other agencies such as the EAC, Stringent Regulatory Authorities (SRAs), Pharmaceutical Inspection Convention (PIC), and the WHO Pre-Qualification Programme (WHO-PQ) (Table 4).

### Functional quality management system

The International Standards Organization (ISO) certification is a globally recognized accreditation of the maturity and functionality of the QMS of an organization. The EAC MRH Projects advocates for implementation of QMS to facilitate delivery of quality and consistent services to customers. The NMRAs in Zanzibar, Burundi, Rwanda and Kenya had no QMS in place during baseline survey, the TMDA was ISO 9001:2008 certified and the NDA had initiated the ISO certification process. Evaluation of QMS implementation data from the EAC Partner States NMRAs indicated different levels of implementation as indicated in Table 5. However, currently the NMRAs in Kenya, Tanzania mainland, Uganda and Zanzibar are ISO 9001:2015 certified, while Rwanda and Burundi NMRAs are working towards the certification [36].

**Table 5:** Status of the Quality Management System in the EAC Partner States NMRAs (2011/12–2014/15)

	National Medicine Regulatory Agency					
	Burundi	Kenya	Rwanda	Tanzania	Uganda	Zanzibar
**Indicator 12:** Implementation of EAC QMS in the NMRA based on ISO-9001 standard	No	Yes (2011)	No	Yes (2014)	Yes (2014)	Yes
**Indicator 13:** ISO-9001 Certification of NMRA medicines regulatory system	No	No	No	Yes (2010)	No	No

NB: Year in brackets indicates the time from which the indicator started to apply

### Functional information management systems

One of the objectives of the EAC MRH is to have all the Partner States’ NMRAs implementing a common IMS for medicines registration, which is linked in all Partner States and the EAC Secretariat. A robust IMS is key for supporting the technical aspects of medicines regulation, to facilitate efficiency and effectiveness of business processes, improve transparency, facilitate decision-making process, sharing and exchange of information among NMRAs and stakeholders, and timeliness of approval of registration decisions. During the baseline survey, the TMDA had an integrated system which was locally developed whereas the PPB used SIAMED®. The NDA, on the other hand, used a combination of SIAMED®, ACCESS® and a locally developed software. Burundi had a manual IMS, which was not functioning, ZFDA had a functional manual registry and Rwanda had both a manual and an electronic system. The method of capturing information in NMRAs in the EAC Partner States was variable and unreliable. The electronic systems were not user friendly, difficult to integrate with other IMS platforms, could not automatically generate or print certificates or be linked to websites. The existing manual systems were labour intensive.

**Fig. 2:**
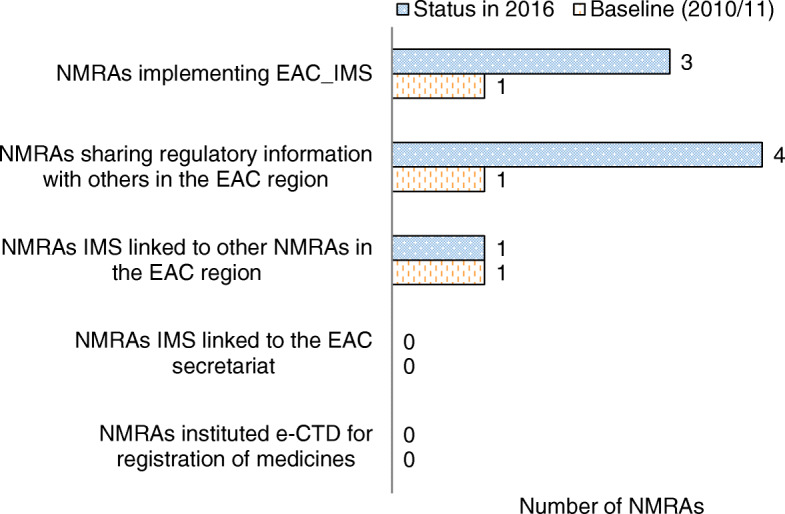
Status of Implementation of Information Management Systems in the EAC Partner States NMRAs (2010/11–2015/2016)

A comparison of IMS implementation status with baseline data shows a significant improvement in all countries, with Burundi and Zanzibar moving from manual to electronic systems. All the EAC NMRAs are currently using a harmonized IMS whereby four out of five NMRAs reported sharing regulatory information amongst each other as indicated in Fig. 2 and Table 6.

**Table 6:** Status of implementation of different key modules by the NMRAs under Information Management Systems (IMS) category in by 2015

	National Medicine Regulatory Agency						
	Burundi	Kenya	Rwanda	Tanzania	Uganda	Zanzibar	EAC Secretariat
**Indicator 14: Status of key modules in Implementation of requirements for an integrated IMS**							
Premises	✓	✓	✓	✓	✓	✓	✕
Products	✕	✓	✓	✓	✓	✓	✕
GMP	✓	✓	✓	✓	✓	✓	✕
Inspection	✕	✓	✓	✓	✓	✓	✕
Import and Export	✕	✓✓	✓✓	✓	✓✓	✕	NA
Finance Module	✓✓✓	✓✓✓	✓	✓✓✓	✕	✕	NA
Report Module	✓✓✓✓	✓✓✓✓	✓✓✓✓	✓✓✓✓	✓✓✓✓	✓✓✓✓	✓✓✓✓

Key: ✓ = fully functional; ✓✓ **=** fully functional and integrated to National Revenue Authority; ✓✓✓ = fully functional and integrated to e-banking and/or Mobile money; ✓✓✓✓ = fully functional and reports customized according to the national and regional needs; ✕ = non-functional; NA = not applicable (Source: EAC Secretariat MRH Project Progress Report, 2015

## Discussion

The current environment of increased globalization imposed by the geo-economic-political situation warrants an advocacy for cooperation and harmonization among NMRAs across the world as basis for building and strengthening regulatory capacity. This is especially important given the fact that harmonization initiatives support regulators’ mandate to promote and protect public health when backed with clear governance structures and regulatory frameworks [37].

Medicines policies and laws serve as the foundation for effective regulation of medical products. A comparative study of medicines laws in the EAC region revealed that medicines laws exist in all EAC Partner States with varying legal provisions, key regulatory functions, and practices. These potentially affect availability of good quality, safe and efficacious medicines needed on the market [35]. In order to ensure effective regulation of medical products, convergence of regulatory practices through streamlining the existing legal frameworks is essential [35]. In this regard, it is important for countries to consider domestication of the AU Model Law on Medical Products Regulation which has the necessary provisions for a country to have a robust regulatory system such as core regulatory functions (as recommended by WHO), some level of autonomy, NMRA’s participation in harmonization initiatives and collaboration with other agencies. The increased advocacy under the EAC MRH Project has helped in creating awareness on the part of policy makers and politicians on the importance of investing in medical products regulation with subsequent improvement in policy and legal frameworks in all the countries of the EAC region.

Regulatory harmonization facilitates pharmaceutical companies’ submission of a single set of the dossier to several different countries with subsequent reduction of costs. Harmonization processes are also beneficial for public health as they facilitate open-minded technical discussions and the exchange of ideas and experience among regulators from different countries [38]. The EAC regulatory harmonization experience has enhanced regulatory science by increasing the rigour of the review process, with subsequent higher quality standards than national procedures. In turn, this has reduced the overall regulatory burden and led to less duplication of efforts [36, 39, 40]. These contribute to the strengthening of the capacity of the NMRAs in expedition of the assessments of priority medicines and filtering out of substandard or falsified products [38]. Sound, efficient, effective and transparent regulatory systems are as well important for the promotion of investment in pharmaceutical sector and socioeconomic advancement [11].

The decision to harmonise guidelines for registration of medicines and GMP inspections in the EAC region has simplified the process for manufacturers intending to lodge an application in any of the countries [59]. In addition, reliance mechanisms for registration of medicines employed by PPB, TMDA, ZFDA and NDA; reliance mechanisms for GMP inspection implemented by RFDA, NDA and ZFDA; and appropriate governance mechanisms to support sound and timely regulatory decisions by NMRAs are attributed to the improved efficiencies observed in these countries. For example, there has been a considerable improvement in the NMRA governance frameworks with a subsequent establishment of autonomous agencies in Kenya, Tanzania mainland, Rwanda, Uganda and Zanzibar.

The attainment of ISO certification by NMRAs is another factor that can be related to improved regulatory efficiency. With a resultant increase in the number of received applications as observed in Kenya, Tanzania mainland and Uganda. The ISO certification will also increase confidence in the quality of NMRAs work, increasing trust and facilitate reliance on registration and inspection processes as viewed by peer agencies in the region and across the continent [36]. It is important to note that ISO certification of the NMRAs is one of drivers of a robust medicines regulatory system. Similar means in place include being ranked by the WHO – GBT for evaluation of national regulatory systems. This was demonstrated by the TMDA by achieving the WHO – Maturity level 3 in 2018, becoming the first African NMRA to do so [41]. According to the WHO, the attainment of Maturity level 3 indicates that an agency has a stable well-functioning and integrated system of oversight for medical products. Further, it is of essence to recognise the progress made by the PPB, NDA and ZFDA, considering they had no QMS in place during the baseline survey. Rwanda is moving toward the attainment of the ISO 9001:2015 certifications while the NMRA in Burundi implemented quality objectives and standard operating procedures for some regulatory activities, including medicines evaluation and registration [36].

Furthermore, an operating IMS that links all Partner States is essential for improving efficiency as it assists the EAC Secretariat and NMRAs in tracking the progress of applications in their own as well as neighboring countries. All EAC Partner States now have a functioning IMS, and it is agreeable that this has boosted efficiency and strengthened cross-departmental linkages. The IMS platforms are designed to allow as many processes as possible to be conducted online through a single portal, whereby the PPB is the only agency which allows the electronic submission of dossier applications. Implementation of the EAC Cooperation Framework agreement also requires IMS platform for NMRAs to communicate and share information [36].

Harmonization initiatives are not spared of challenges. One of the challenges experienced by the EAC harmonization initiative is the unfamiliarity with regulatory systems of other NMRAs. This resulted into a lengthy process from development of harmonized guidelines to the actual coming into force in January 2015. The process involved eleven steps to familiarise all the key stakeholders such as regulators and industry with new guidelines and entailed additional costs for sensitization and training [40, 42]. Experience shows that sometimes countries are unwilling to commit to a uniform code due to differences in political economy and ethical systems. In a situation where there are differences in legal frameworks, it may imply that definitions of terms among countries are different (“generic”, “reference product”, “data exclusivity”, “pharmacopoeia”, “variations”, etc.) hence causing delays in the harmonization process. Medicines acceptable for market authorizations by the NMRAs as well as acceptable indications may also vary between countries based on differences in treatment guidelines and therapeutic traditions. Other challenges include differences in technical requirements, for example bioequivalence (BE) requirements for complex products and differences in aspects related to product and Active Pharmaceutical Ingredients (API) (e.g. source, method of manufacture and packaging). Moreover, divergence following joint approval due to separate handling of post-approval changes, variations in assessment timelines based on regulations and differences in data exclusivity/patent rules, are other factors to be well considered when implementing harmonization initiatives [40].

Although some countries have proven to be faster than others in granting marketing approval of products following the regional review process, many applicants are hesitant to use the joint product assessment procedure until efficiency improvements are made. While it has been reported that the regional procedure is of unexpectedly higher quality standards than national procedures, a common frustration is the time taken to receive the actual marketing authorization especially for smaller, less attractive markets [39]. These differences may be due to the diverse nature of governance (whether autonomous agency with its own Board or a department under the Ministry of Health) and execution of roles among the NMRAs, as well as the existence of non-streamlined policies, laws and guidelines in the respective countries. There is a need to further study factors hampering national uptake of joint review process as they affect marketing authorization timelines. Improvements are therefore required for the current EAC processes to meet the vision of harmonization.

Key performance indicators developed as part of this study have helped to identify gaps in the regional harmonization process. While the baseline assessment tool focused on the national regulatory system, legal framework, marketing authorization and regulatory inspection, the indicators tool developed during this study provided more insights into the interactions between NMRAs. These are mainly through regional processes like implementation of regional harmonised guidelines, execution of joint regulatory activities and subsequent uptake of the outcomes of the regional joint review and inspection processes.

Harmonizing regulatory standards across countries, work and cost-sharing arrangements, collaboration between NMRAs, as well as advocacy and information campaigns are key aspects in addressing the existing regulatory limitations in and across countries. The AMRH provides a platform to support RECs, Regional Health Organizations (RHOs) and Member States, in harmonizing medicines regulation with a view to build and strengthen regulatory capacity. These are achieved through mobilizing interested governments, donors and other stakeholders to invest in medicines regulation [18, 29]. The AMRH Initiative also serves as a foundation for the establishment of the African Medicines Agency (AMA) as enshrined in the AU Executive Council Decision EX.CL/872(XXVI) of January 2015 [20, 43]. It is expected that these initiatives will accelerate research and development of new or improved medical interventions for Poverty-Related Neglected Diseases (PRNDs), provide an enabling environment for local manufacturing, and contribute towards the AU Agenda 2063 and the global 2030 SDGs [18, 20].

### Way forward for the initiative

Review of the EAC MRH Project in 2017 identified three key challenges including limited access to public information about the process; limited information flow from regulators to industry during the joint assesment process; and a significant lag time between a joint assessment recommendation and national registration decisions.

As a result of these challenges, the EAC Partner States came up with a package of solutions. First, the NMRAs of the Partner States agreed on a cadre of Regional Technical Officers (RTOs) who are responsible for the day-to-day management of joint activities and recommending programmatic changes to the initiative’s Steering Committee. The Partner States also agreed on implementing the Cooperation Framework Agreement for the NMRAs, as also approved by the EAC’s Council of Health Ministers in May of 2018. This serves as an intermediary step toward a binding mutual recognition agreement between all Partner States. The cooperation framework will facilitate implementation of joint assessment or inspection decision and ensure that regulatory decisions are made in a timely manner and honoured throughout the region. On funding the Initiative, the region will introduce a framework for contributions from the Partner States’ NMRAs. This includes a coordination fee to support regional assessment and inspection processes to complement donor funding. The long-term goal is to establish a semi-autonomous EAC Medicines Agency which will ensure that the program continues to grow and improve [44].

The process of widening the scope of medical products has started with harmonization of regulation of in vitro diagnostics (IVDs), and medical devices with a view to include vaccines, biologics and biosimilars. Regulatory activities will also be expanded to cover clinical trials oversight, safety and quality surveillance initiatives. Furthermore, the initiative has welcomed the South Sudan’s new NMRA towards addressing the regulatory challenges as the youngest member of the EAC [44].

### Study limitations

Missing some of the data from some of the NMRAs led to the lack of a complete regional picture in several aspects of this study.

## Conclusion

The improved operational efficiencies with subsequent faster and more consistent review and approval process is a result of the regulatory harmonization, which has facilitated faster availability of products in the EAC market. It is expected that regulatory and product development costs will decrease and the industry submission practices will be aligned with fewer parallel registrations. Moreover, the resultant mutual learning and consistency in applying international guidelines for registration of medicines or inspection of manufacturing sites are anticipated.

The study has shown improved regulatory capacity in the EAC Partner States at varying degrees and timelines. The EAC Partner States have strived to put in place comprehensive policies and legal frameworks to support regulation of medical products with subsequent use of harmonised regional guidelines. Quality management system and information management systems have shown to be necessary tools for improving efficiency of regulatory processes. Moreover, the improved work-sharing through shared knowledge and skills among NMRAs has resulted in faster regulatory approvals and improved availability of safe, efficacious, and good quality medicines.

The developed indicators tool serves as basis for evaluation of regulatory harmonization networks on the African continent and beyond. We expect that the gaps identified, and lessons learnt from using the indicators tool in this study can be used as basis to refine it for further use.

## Data Availability

All relevant data are within the manuscript. The data collection instruments, and the datasets used during the current study are available from the corresponding author upon request.

## Abbreviations

ABREMA: *Autorite’ Burundaise de Regulation des Medicaments et des Aliments* (Drug and Food Regulatory Authority of Burundi)
AfCFTA: African Continental Free Trade Area
AMA: African Medicines Agency
AMRH: African Medicines Regulatory Harmonization
API: Active Pharmaceutical Ingredient
AU: African Union
BE: Bioequivalence
CTD: Common Technical Document
EAC: East African Community
ECCAA: Economic Community of Central African States
ECOWAS: Economic Community of West African States
GDP: Gross Domestic Product
GMP: Good Manufacturing Practice
IGAD: Intergovernmental Authority on Development
IMS: Information Management System
ISO: International Standards Organization
IVDs: In vitro Diagnostics
NDA: National Drug Authority
NMPs: National Medicines Policies
NMRA: National Medicines Regulatory Authority
PIC: Pharmaceutical Inspection Convention
PMPA: Pharmaceutical Manufacturing Plan for Africa
PPB: Pharmacy and Poisons Board
PRNDs: Poverty-Related Neglected Diseases
QMS: Quality Management Systems
RECs: Regional Economic Communities
RFDA: Rwanda Food and Drugs Authority
RHOs: Regional Health Organizations
RTOs: Regional Technical Officers
SDGs: Sustainable Development Goals
SRAs: Stringent Regulatory Authorities
TMDA: Tanzania Medicine and Medical Devices Authority
UHC: Universal Health Coverage
WHO: World Health Organization
WHO-GBT: World Health Organization Global Benchmarking Tool
WHO-PQ: World Health Organization Pre-Qualification Programme
Zazibona: Zambia, Zimbabwe, Botswana and Namibia
ZFDA: Zanzibar Food and Drug Authority
ZFDB: Zanzibar Food and Drugs Board
